# An efficient pyramid scene parsing network with multi-scale feature fusion for liver segmentation in magnetic resonance imaging

**DOI:** 10.3389/fmed.2026.1792734

**Published:** 2026-05-11

**Authors:** Monisha Perumal, Jagadeesh Gopal

**Affiliations:** School of Computer Science Engineering and Information Systems, Vellore Institute of Technology (VIT), Vellore, Tamil Nadu, India

**Keywords:** EfficientNetB0, liver, multi-scale feature fusion, PSPNet, Resnet50, skip connections

## Abstract

**Background:**

Recent lifestyle changes have led to an increase in the number of liver disease cases, making accurate liver segmentation increasingly important for clinical applications. However, manual segmentation is time-consuming, operator-dependent, and can vary between experts, creating a need for reliable automated approaches.

**Methods:**

In this study, a PSP-EffB0-MSFF model is proposed for 2D liver segmentation from abdominal MRI images. EfficientNetB0 replaces the ResNet50 backbone in PSPNet to reduce computational cost, and a multi-scale feature fusion module is incorporated into the skip connections to improve feature representation. The model is evaluated using two datasets: T1-weighted images from DLDS and T2-weighted images from CirrMRI600+.

**Results:**

On the DLDS dataset, the model achieves an intersection over union of 0.905 ± 0.038 and a Dice score of 0.913 ± 0.09, along with a Hausdorff distance of 7.31 ± 3.91 and an average sym metric surface distance of 2.66 ± 3.06. On the CirrMRI600+ dataset, it achieves an intersection over union of 0.86 ± 0.01 and a Dice score of 0.90 ± 0.02, with a Hausdorff distance of 6.20 ± 0.60 and an average symmetric surface distance of 9.80 1 1.50 at the patient level. The model requires 14.91 GFLOPs.

**Conclusion:**

Overall, the proposed PSP-EffB0-MSFF model provides reliable segmentation results on CirrMRI600+ and shows consistent performance on DLDS under the current experimental setup.

## 1 Introduction

The liver is an important organ in the human body that performs many functions, such as detoxification of hormones, production of biochemicals necessary for digestion, purification of blood by removing its waste products, regulation of blood clotting, and protein synthesis (1). Many cases of liver disease are associated with hereditary factors, weakened immunity, excessive alcohol consumption, and changes in dietary habits. Liver diseases include hepatitis, cirrhosis, fatty liver disease, and cancer. Viruses, alcohol, medicines, toxins, and autoimmune diseases can all cause liver inflammation and hepatitis. Cirrhosis is a late stage of liver scarring that is, usually induced by chronic hepatitis or

heavy alcohol use. Individuals who do not consume alcohol may develop non-alcoholic fatty liver disease (NAFLD). It is associated with obesity, diabetes, and metabolic syndrome. Alcoholic liver disease includes fatty liver diseases, alcoholic hepatitis, and cirrhosis (2). In 2015, the WHO projected that 257 million individuals were chronically infected with HBV. Patients with chronic HBV infection are at risk of developing liver diseases, such as cirrhosis and HCC (3). Precise liver segmentation is essential in various medical procedures, such as calculating liver volume for surgical planning, evaluating tumor load, assessing treatment effectiveness, and tracking disease progression in conditions, such as cirrhosis and hepatocellular carcinoma. The manual outlining of the liver in MRI images is labor-intensive and subject to inter-observer variability. Consequently, automated liver segmentation techniques can aid radiologists by providing consistent quantitative data and alleviating the burden of manual annotation. Ultrasound (US), Computed Tomography (CT), Magnetic Resonance Imaging (MRI) and elastography are the most widely used imaging modalities for diagnosing and assessing liver disorders (4). Among these imaging methods, MRI is the most comprehensive non-invasive technique, distinguished by its superior contrast resolution for soft tissues, lack of ionizing radiation, and capability to utilize contrast-enhanced techniques to detect liver lesions, fibrosis, and cirrhosis (5). Currently, illnesses are diagnosed using Machine Learning, Deep Learning, and other methods. This saves detection time, reduces the cost of identification devices, and aids in the identification of tiny details. Liver MRI scans are complicated by the irregular shape of the liver in the imaging area. Medical professionals generally prefer MRI for the diagnosis of liver pathology. A new cascaded network architecture was developed for the segmentation of liver tissue from T1-weighted magnetic resonance images (6). As healthcare data becomes increasingly complex, artificial intelligence has emerged as a valuable tool to assist in the diagnosis and management of liver diseases, helping to reduce human error and improve clinical decision-making. (7). A deep learning model, specifically UNet and a modified Residual UNet, were employed to segment the liver and tumor in CT images on the 3Dircadb dataset (8). A semi-supervised framework was introduced to enhance liver segmentation in abdominal MRI scans, even with a limited amount of labeled data. This framework incorporates cross-teaching, pseudo-label generation, and entropy minimization and leverages the capabilities of U-Net and MedNeXt (9).

Investigations of liver tumors using MRI, which involves radiomics and deep learning techniques, are becoming more prevalent. The manual process for distinguishing normal liver tissues from tumorous tissues is limited. Automatic segmentation of liver tumors and segments has been achieved using UNet++ (19). A liver tumor segmentation framework based on a Multi-level Dual Aspect Self-Attention Guided Transformer (MDA-SGT) was introduced, where a Probabilistic Fused Wiener Filter was used for preprocessing, and Res2Net was employed for feature extraction from CT and MRI images (20). The diseased area in the ultrasound image was isolated using an enhanced active contour-based segmentation approach (21). The initial liver segmentation process involved a two-step cascade procedure. The initial step outlines the focus area, on which the subsequent step relies as its input. This step reduces the classification load in the second step by decreasing the number of voxels that must be processed, thereby enabling more precise liver segmentation (22). A modified version of the UNet-60 model was used to detect and classify liver disease tumors. Tumors identified on computed tomography images have been categorized as either metastatic or cholangiocarcinoma (23). The CAML-PSPNet network was developed to improve segmentation accuracy by incorporating a coordinate attention mechanism with a mixed loss function. This model utilized MobileNetV2 as its backbone, facilitating lightweight feature extraction (24). A spatio-temporal decoupling network (STD-Net) was proposed to separate spatial feature extraction using a shared-weight 3D encoder and temporal dynamics modeling through a transformer-based module for multiphasic CT and MRI analysis (25). Despite recent advances in deep learning, liver segmentation from MRI remains challenging because of limited global context modeling in CNN-based models, the high computational cost of transformer-based approaches, and ineffective multiscale feature integration in conventional architectures, such as PSPNet and U-Net, which pass skip connections directly to the decoder without feature enrichment. As shown in Table 1, most existing studies have focused on CT imaging, leaving MRI-based segmentation relatively underexplored despite its superior soft-tissue contrast and suitability for repeated follow-up imaging in chronic liver disease. Therefore, studies from other imaging modalities and related tasks are included for contextual comparison, although they are not directly equivalent to MRI-based whole-liver segmentation. Clinically, inaccurate liver boundary delineation affects liver volumetry, preoperative surgical planning, transplantation assessment, and cirrhosis monitoring, where radiologists currently depend on time-consuming manual annotation, which is subject to inter-observer variability. Although deep learning methods have shown strong performance for liver segmentation in CT images, their application to MRI remains challenging. This is mainly due to the lower soft-tissue contrast, intensity variations, and difficulty in distinguishing liver boundaries from nearby organs (4, 5). Consequently, models designed for CT data do not always transfer well to MRI. Conventional U-Net-based approaches pass multi-scale features directly through skip connections without additional refinement, which can affect the boundary accuracy in MRI (19, 22). Transformer-based models, on the other hand, improve global context modeling but often come with higher computational costs, making them less practical for large MRI datasets (11). Although PSPNet captures multi-scale contextual information, it mainly relies on high-level encoder features and makes limited use of intermediate representations that are important for accurate boundary recovery. In addition, commonly used backbones, such as ResNet-50, increase the computational cost, whereas lightweight alternatives may not fully preserve the feature representation capacity (12, 24). To address these issues, the proposed PSP-EffB0-MSFF model combines an EfficientNet-B0 backbone with MSFF modules applied to skip the connections. This design allows for better integration of multi-scale features and improves the representation of boundary-related information across different resolutions, which is particularly important for MRI-based liver segmentation. Segmentation was performed on 2D MRI slices. The following is an outline of the main research contributions:

- A PSPNet framework was developed with EfficientNetB0 as a lightweight encoder. This backbone is combined with a pyramid pooling module at the bottleneck to support multi-scale contextual aggregation.
- Four skip connections were derived from the intermediate blocks of EfficientNetB0 to connect encoder features at different spatial resolutions to the decoder.
- The MSFF block was proposed to handle every skip link via three parallel branches comprising a 1 × 1 convolution, 3 × 3 convolution, and 3 × 3 dilated convolution, along with a channel attention mechanism and residual connections.
- The decoder merges the MSFF-enhanced skip connections throughout the four progressive upsampling stages, which are combined with hierarchical channel reduction, where each stage applies concatenation, channel reduction, and feature refinement operations.

**Table 1:** Summary of existing liver segmentation studies.

References	Year	Task (modality)	No. of images	Dataset sources	Methods	Limitations
Patel et al. (10)	2024	Liver segmentation (MRI, T1-weighted)	819 MRIs	72 patients (Duke), 34 (MD Anderson), 71 (Houston Methodist), 58 (Bourgogne, France), 57 (China), 20 (Turkey)	nnUNet, PocketNet, Swin UNETR	Model's performance was evaluated only on T1 images.
Haensch et al. (11)	2022	Liver segmentation (MRI)	107 DCE-MRI images	Ethics-approved imaging from Sächsische Landesärztekammer	3D U-Net with multimodal training	single source dataset
Marti-Aguado et al. (12)	2022	Liver segmentation (MRI)	165 patients	Valencia, Spain	CNN-based whole-liver deep learning segmentation with PDFF and R2* measurements	The model is not able to exclude small vessels within the segmented volume
Giri et al. (13)	2024	Liver segmentation (CT)	100 CT scan images	Medical Segmentation Decathlon	U-Net	Required high computational resources
Jagarapu et al. (14)	2025	Liver segmentation (MRI)	T1–310 patients, T2–318 patients MRI images	CirrMRI600+ dataset	Dual-Branch-Network3D	Uses a single dataset for evaluation.
Gross et al. (15)	2024	Liver segmentation with volumetry and radiomics (MRI)	470 patients (internal), 20 patients (external)	Beaujon Hospital in Clichy, France and LiverHccSeg	3D DCNN	Only used T1-images
Fallahpoor et al. (16)	2024	Liver and liver lesion segmentation (MRI)	MRI collected from 128 patients.	private dataset	Modified 3D U-Net	Fail to identify a small lesion
Prencipe et al. (17)	2022	Liver segmentation (CT)	443 portal venous phase CT scans	MSD 08 dataset	2.5D CNN (V-Net)	single-source dataset
Sharma et al. (18)	2023	Gastrointestinal segmentation (MRI)	85 patients MRI scans	Wisconsin-Madison in Madison, Wisconsin	Pyramid Scene Parsing Network (PSPNet)	Limited patient diversity

## 2 Methodology

### 2.1 Dataset

The DLDS dataset consists of T1-weighted MRI scans from 95 patients, whereas the CirrMRI600+ dataset includes T2-weighted MRI images from 318 patients. Because the proposed approach operates on 2D inputs, the DLDS volumes were first converted into axial slices. As the two datasets correspond to different MRI modalities (T1 and T2), noticeable differences in image intensity and contrast were observed. Both datasets were split at the patient level into training (70%), validation (10%), and testing (20%) sets for analysis. During training, a fixed number of slices were selected from each patient using uniform sampling across the available slices (up to 50 slices per patient for DLDS and up to 25 slices per patient for CirrMRI600+). These slices were fixed prior to training and reused across all runs to ensure reproducibility. During validation and testing, all available slices from each patient were used to provide a comprehensive evaluation. The total number of slices per subset depends on the number of slices available for each patient and therefore varies across the datasets. The distribution of patients across the three subsets is shown in Table 2.

**Table 2:** Patient-level dataset split.

Dataset	Split	No. of patients
DLDS	Train	66
DLDS	Validation	10
DLDS	Test	19
CirrMRI600+	Train	222
CirrMRI600+	Validation	32
CirrMRI600+	Test	64

### 2.2 Preprocessing

The DLDS and CirrMRI600+ datasets were acquired under different scanning protocols, resulting in variations in spatial resolution across patients. To ensure a consistent input format, all images and corresponding masks were resized to 256 × 256 pixels prior to model input. The EfficientNetB0 backbone was pretrained on RGB images; each grayscale MRI slice was replicated across three channels to form a 256 × 256 × 3 input tensor. Pixel intensities were normalized using the EfficientNet preprocessing function, which rescales values to the range of [–1, 1] to match the expected input distribution of the pretrained weights. The masks were resized to the same spatial resolution and binarized, with liver regions assigned a value of 1 and the background assigned a value of 0. To manage the computational cost during training, a fixed number of slices were selected per patient (up to 50 slices for DLDS and 25 for CirrMRI600+) using uniform sampling across available slices. During evaluation, all slices per patient were used, including both liver-containing and background-only slices, thereby providing a consistent and unbiased assessment of segmentation performance.

### 2.3 PSP-EffB0-MSFF

A lightweight Multi-Scale Feature Fusion (MSFF) module was developed in conjunction with the original PSPNet-EfficientNetB0 architecture for the segmentation of the liver in MRI images. The architecture proposes a Pyramid Pooling Module (PPM) at the bottleneck for the aggregation of multi-scale contexts and an efficient encoder backbone, EfficientNetB0, replacing the traditional ResNet.

**Figure 1:**
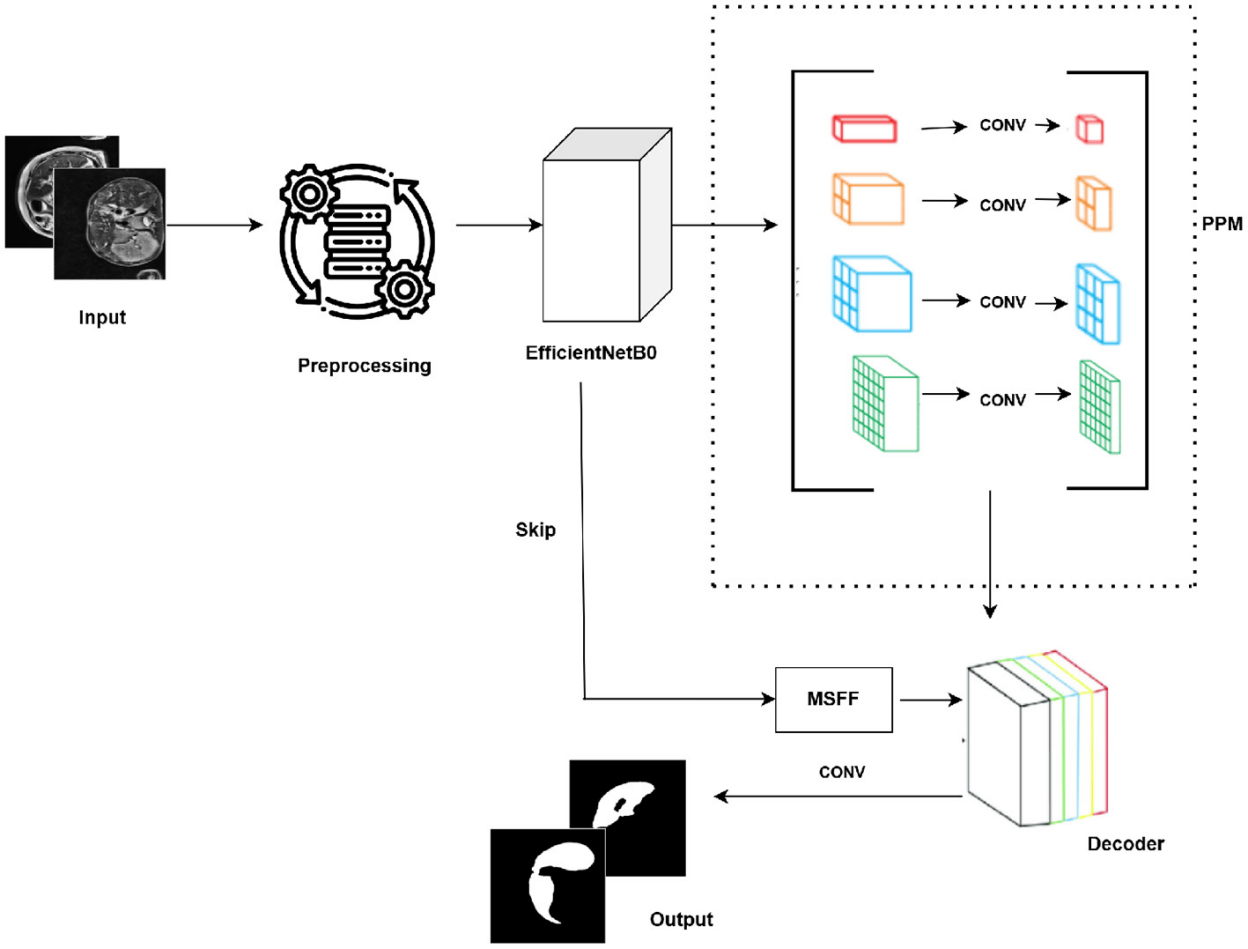
Proposed PSP-EffB0-MSFF framework.

The encoder extracts features at multiple levels, from which four skip connections are obtained at different spatial resolutions. Each of these skip features is further refined using the MSFF module, which combines multi-scale convolutional operations (1 × 1, 3 × 3, and dilated 3 × 3) with channel attention and a residual connection. In the decoder, the feature maps are progressively upsampled, and the MSFF-refined skip connections are concatenated at each stage to improve spatial detail recovery. The channel dimensions are gradually reduced during this process. The overall workflow of the proposed model is illustrated in Figure 1.

**Figure 2:**
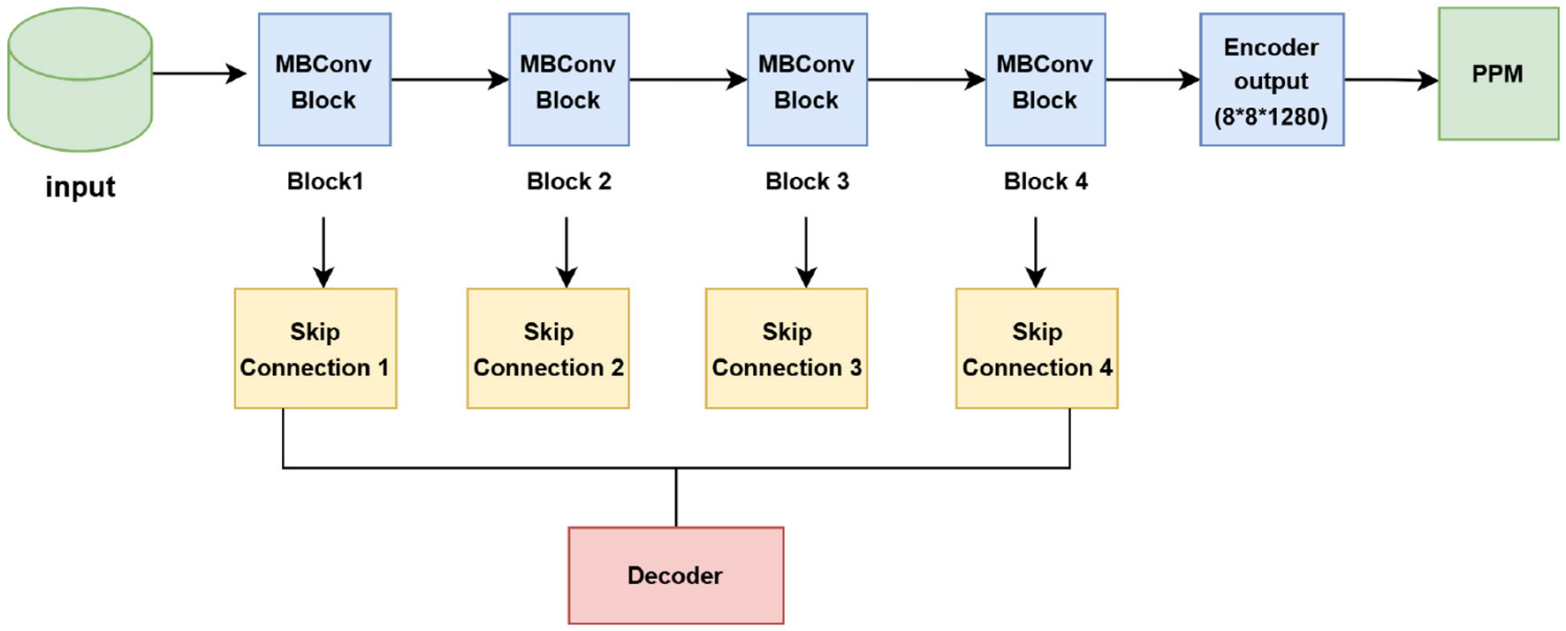
Encoder.

Figure 2 illustrates the working flow of the encoder. The EfficientNetB0 architecture is the encoder backbone that extracts the features of the liver MRI image (256 × 256 × 3) at different levels of abstraction. The model initially extracts low-level features such as edges and corners, and progressively learns more complex patterns corresponding to organ structures, tissue characteristics, and regions of the liver. The EfficientNetB0 network processes the input image through a succession of Mobile Inverted Bottleneck Convolution (MBConv) blocks together with Squeeze-and-Excitation (SE) mechanisms, which help the network effectively detect the local features and global context. Intermediate feature maps at multiple resolutions (128 × 128, 64 × 64, 32 × 32, and 16 × 16) are extracted and retained as skip connections at key stages of the encoder. These feature maps were selected to capture multi-scale spatial information from the encoder. These four resolutions were selected based on the internal structure of EfficientNetB0. As the input image propagates through the network, the spatial resolution is progressively reduced at specific MBConv block transitions (Block 2a, Block 3a, Block 4a, and Block 6a), producing feature maps with sizes of 128 × 128, 64 × 64, 32 × 32, and 16 × 16, respectively, for a 256 × 256 input. These feature maps correspond to different levels of feature abstraction: the 128 × 128 map captures low-level spatial details, such as edges and textures; the 64 × 64 and 32 × 32 maps encode mid-level structural information related to organ boundaries and tissue regions; and the 16 × 16 map represents high-level semantic features of the liver. Moreover, the progressive halving of the spatial resolution ensures that each feature map aligns naturally with the decoders corresponding 2 × upsampling stages. This consistent alignment enables efficient multi-scale feature fusion without requiring additional interpolation, making these stages well-suited for skip connections in the proposed architecture. These connections preserve vital spatial data and multi-scale features that would otherwise be lost during down-sampling and are then coupled with the features of the decoder to provide precise localization and delineation of the boundaries of the liver in the final segmentation mask. The deepest features at the bottleneck (8 × 8) are subjected to Pyramid Pooling to obtain multi-scale contextual information before being sent to the decoder. At each encoding stage *i*, the feature extraction process can be defined as follows:


$$\begin{array}{l} F_{i}=EncoderBlock_{i}\left(F_{i-1}\right) \end{array}$$ (1)

In Equation 1, *Fi*−1 represents the input feature map from the previous stage, and *F*_*i*_ denotes the output feature map at the current encoding stage. In Equation 2, each encoder stage produces a skip feature map *S*_*i*_, which is subsequently applied to the decoder. The corresponding skip connections are defined as follows:


$$\begin{array}{l} S_{i}=F_{i} \end{array}$$ (2)

Inside the pyramid pooling module (PPM), the output of the encoder is fed into four pooling branches, each with bin sizes of 1 × 1, 2 × 2, 3 × 3, and 6 × 6. In each branch, the average pooling method is used to extract features at a scale peculiar to it. Subsequently, a 1 × 1 convolution is applied, and the output is upsampled to the original size (8 × 8). The processed outputs are then combined with the original input feature map, consequently a multi-scale representation that is detailed and incorporates the vast context from the 1 × 1 pooling, along with the fine details from the 6 × 6 pooling, is formed. After the pyramid pooling operation, the feature maps were concatenated with the output of the encoder. This concatenated feature map is passed through a convolutional layer with a size of 1 × 1 to reduce the channel size to 512. This is the input to the decoder.

**Figure 3:**
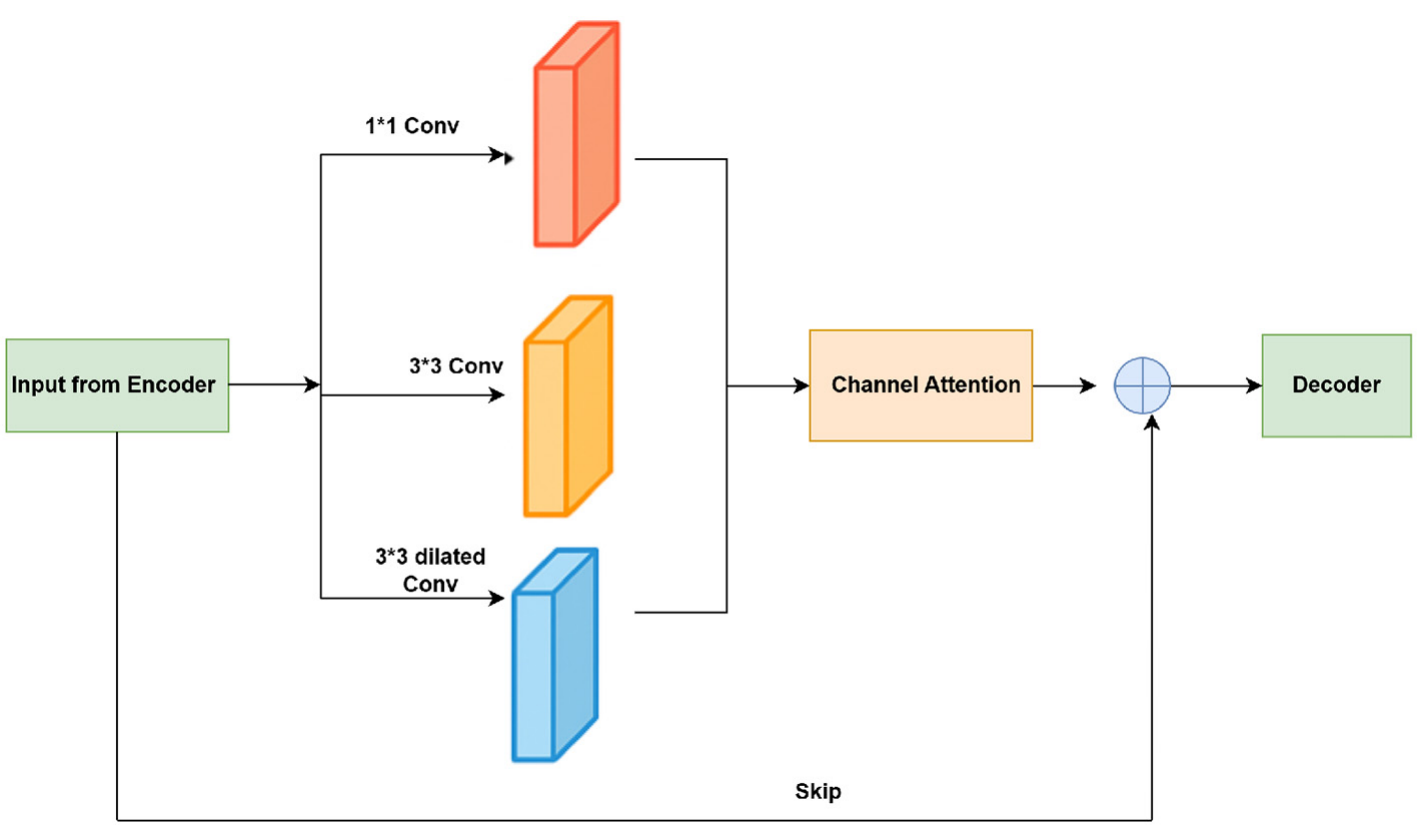
MSFF.

The Multi-Scale Feature Fusion (MSFF) module integrates multi-scale information within each skip connection (Figure 3). The input feature map is simultaneously processed through three parallel branches: a 1 × 1 convolution to capture channel-wise relationships, a 3 × 3 convolution to extract local spatial features, and a 3 × 3 dilated convolution (dilation rate = 2) to capture broader contextual information without increasing computational cost. The three branches operate on the same input feature map. The resulting feature maps are concatenated along the channel dimension and passed through a lightweight channel attention mechanism comprising global average pooling followed by two fully connected layers. The generated attention weights, obtained through a sigmoid activation, emphasize the most informative feature channels. A residual connection is applied by adding the original input feature map to the attention-refined features, which helps to preserve spatial information and improve the gradient flow during training.

In Equation 3, the multi-scale branches are defined as follows:


$$\begin{array}{l} F_{1}={\text{Conv}}_{1\times 1}\left(F_{in}\right),\\ F_{2}={\text{Conv}}_{3\times 3}\left(F_{in}\right),\\ F_{3}={\text{Conv}}_{3\times 3,d=2}\left(F_{in}\right), \end{array}$$ (3)

In Equation 4, the concatenated feature map is expressed as follows:


$$\begin{array}{l} F_{cat}=\left[F_{1}∥F_{2}∥F_{3}\right] \end{array}$$ (4)

where [∥] denotes channel-wise concatenation. The attention mechanism is defined as follows:


$$\begin{array}{l} M=\sigma \left(W_{2}\delta \left(W_{1}\text{GAP}\left(F_{cat}\right)\right)\right), \end{array}$$ (5)

In Equation 5, GAP(·) denotes Global Average Pooling, *W*_1_ and *W*_2_ represent the fully connected layer weights, δ denotes the ReLU activation function, and σ denotes the sigmoid activation function. In each MSFF block, each of the three parallel branches produces an equal number of output channels, which are set to the same dimension as the input feature map. The concatenated output *F*_*cat*_ therefore has three times the input channel dimension, which is then reduced to the original channel size through a channel attention mechanism. For the EfficientNetB0 encoder, the channel dimensions of the skip features were 96, 144, 240, and 672 at resolutions 128 × 128, 64 × 64, 32 × 32, and 16 × 16, respectively. Accordingly, the concatenated MSFF feature maps had 288, 432, 720, and 2016 channels, which were then reduced to their original dimensions using the channel attention mechanism.

Finally, the MSFF output is computed as follows:


$$\begin{array}{l} F_{out}=W_{r}F_{in}+\left(M⊙F_{cat}\right) \end{array}$$ (6)

In Equation 6, ⊙ denotes element-wise multiplication. A 1 × 1 convolution (*W*_*r*_) is applied to project *Fin* to the same channel dimension as *Fcat*, enabling valid residual addition. The fused feature map is then passed to the decoder.

**Figure 4:**
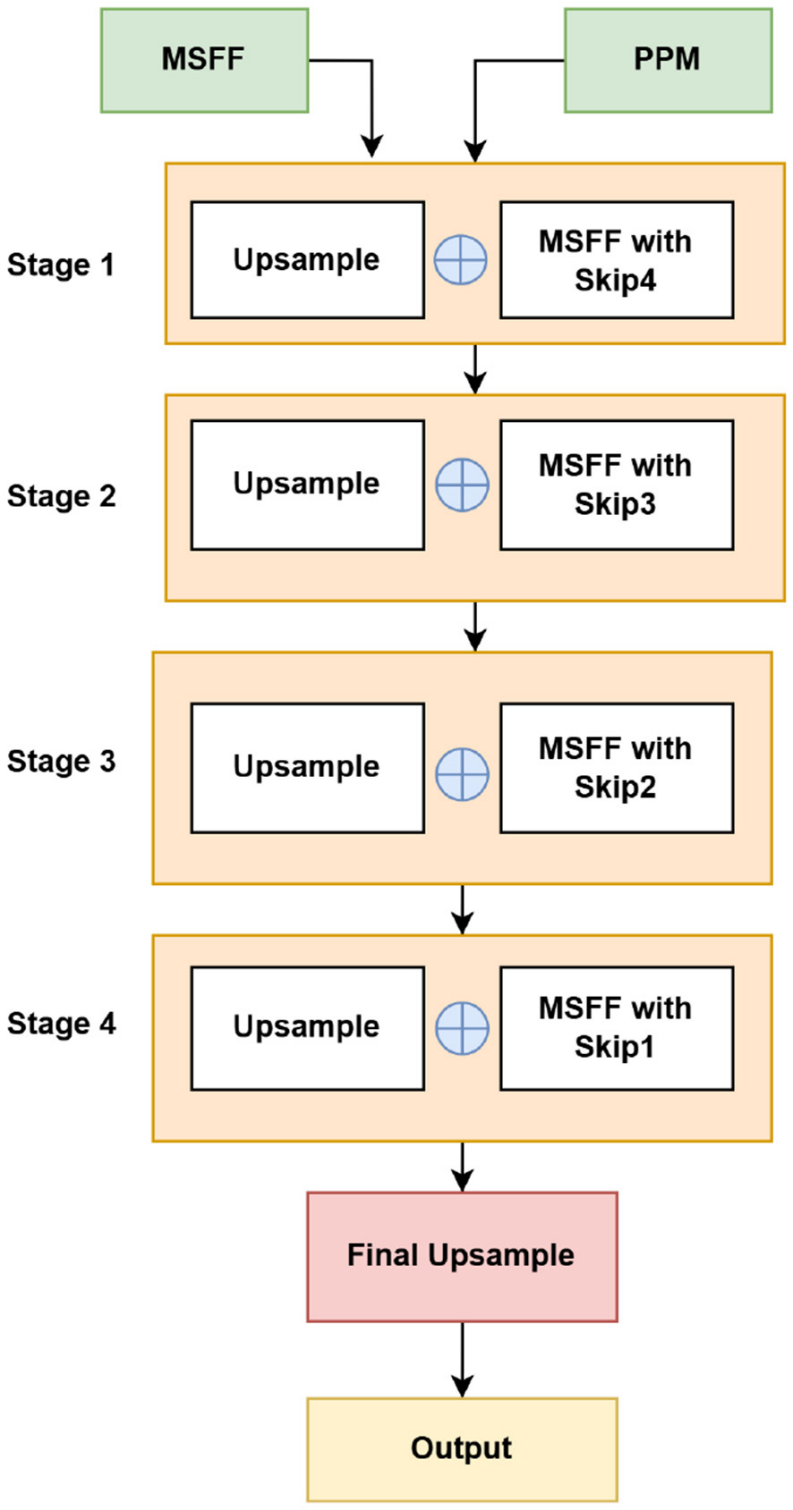
Decoder.

The output of the Pyramid Pooling Module (PPM) is used as the input to the decoder (Figure 4). The decoder gradually upsamples the feature map from 8 × 8 to 256 × 256 through five stages, where each stage doubles the spatial resolution (8 × 8 → 16 × 16 → 32 × 32 → 64 × 64 → 128 × 128 → 256 × 256). At each stage, the upsampled features are combined with the corresponding encoder features, which are enhanced by the MSFF module, through skip connections. The fused features are then refined using convolutional layers. To balance computational efficiency and feature preservation, the channel dimension is gradually reduced at each decoding stage, decreasing from 512 to 256, then to 128, 64, and finally to 32. A final 1 × 1 convolution with a sigmoid activation function is used to generate the segmentation mask. The decoder process is defined as follows:


$$\begin{array}{l} D_{i}=\text{Refine}\left(\text{Concat}\left({\text{Up}}_{2}\left(D_{i-1}\right),\text{MSFF}\left(S_{i}\right)\right)\right),\;i=1,2,3,4 \end{array}$$ (7)

In Equation 7, *D*_*i*_ represents the decoder feature at stage *i*, Up_2_(·) denotes 2 × upsampling, *S*_*i*_ is the encoder feature refined through the MSFF module, and Refine(·) fuses and smooths the concatenated features through a 3 × 3 convolution followed by Batch Normalization and ReLU activation. The initial four stages of the decoder utilize MSFF-enhanced skip connections from the encoder. The final decoding stage performs upsampling without a skip connection to restore the full spatial resolution. At each decoding stage, the refine operation consists of a single 3 × 3 convolution followed by Batch Normalization and ReLU activation, with the output channel dimension reduced progressively (512 → 256 → 128 → 64 → 32).

The final segmentation output is computed as


$$\begin{array}{l} M=\sigma \left({\text{Conv}}_{1\times 1}\left(D_{5}\right)\right), \end{array}$$ (8)

In Equation 8 Conv_1 × 1_ reduces the channel dimension and σ(·) denotes the sigmoid activation function that generates the final mask *M*. The pseudocode for the proposed model is concisely provided above. It encompasses five essential steps: data preprocessing, encoder feature extraction, PPM, MSFF, and decoder restoration for pixel-wise segmentation. The detailed procedures are discussed in the preceding sections. Table 3 summarizes the architecture of the proposed PSP-EffB0-MSFF model. The encoder reduces the input image size from 256 × 256 to 8 × 8 while increasing the feature depth. The PPM is used to capture contextual information at multiple scales and reduces the feature channels to 512. The decoder then restores the spatial resolution through a series of upsampling stages. At each stage, MSFF-enhanced skip connections are used to improve feature refinement. The number of channels is gradually reduced from 512 to 32 to maintain computational efficiency. Finally, a 1 × 1 convolution layer with sigmoid activation is used to generate the segmentation mask.

**Table 3:** Architecture of the proposed PSP-EffB0-MSFF model.

Stage	Resolution	Operation	Channels
Encoder output	8 × 8	EfficientNetB0	1,280
PPM output	8 × 8	Pyramid Pooling + 1 × 1 Conv	512
Decoder stage 1	16 × 16	Upsample + MSFF (skip4) + Concat + Conv	512
Decoder stage 2	32 × 32	Upsample + MSFF (skip3) + Concat + Conv	256
Decoder stage 3	64 × 64	Upsample + MSFF (skip2) + Concat + Conv	128
Decoder stage 4	128 × 128	Upsample + MSFF (skip1) + Concat + Conv	64
Decoder stage 5	256 × 256	Upsample + Conv	32
Output	256 × 256	1 × 1 Conv + Sigmoid	1

### 2.4 Implementation details

The proposed model was implemented using TensorFlow and Keras. The EfficientNetB0 backbone was initialized with ImageNet pre-trained weights. Because MRI slices are grayscale, each input image was replicated across three channels to match the expected RGB input format of EfficientNetB0. The entire backbone was kept fully trainable from the start of training to enable task-specific fine-tuning. The model was trained using the Adam optimizer with an initial learning rate of 2 × 10^−4^, a batch size of 16, and a maximum of 25 epochs. A combination of binary cross-entropy and Dice loss was used as the training objective, with equal weighting (λ = 0.5 for each loss component). The learning rate was reduced by a factor of 0.5 if the validation Dice coefficient did not improve for six consecutive epochs, with a minimum learning rate of 1 × 10^−7^. Early stopping with a patience of 12 epochs was applied, and the best model weights were selected based on the highest validation Dice score. Mixed-precision training was employed to accelerate training and reduce GPU memory consumption, while maintaining numerical stability. During inference, a threshold of 0.5 was applied to the sigmoid outputs to generate binary segmentation masks. All experiments were conducted on an NVIDIA Tesla T4 GPU using Google Colaboratory.

Algorithm 1 PSP-EffB0-MSFF.

  Step 1: data preprocessing
  Load MRI images and corresponding masks
  Resize images and masks to 256 × 256
  Normalize images using EfficientNet preprocessing (range [−1, 1])
  Replicate grayscale image across three channels (RGB format)
  Step 2: build encoder with efficientNetB0
  - Load EfficientNetB0 (weights = imagenet, include_top = False)
  - Extract skip connections:
  skip1: 128 × 128 × 96
  skip2: 64 × 64 × 144
  skip3: 32 × 32 × 240
  skip4: 16 × 16 × 672
  encoder output: 8 × 8 × 1, 280
  Step 3: pyramid pooling module (PPM)
  - For each bin size *b* ∈ {1, 2, 3, 6}:
  pooled ← AvgPool(feature/*b*)
  Conv2D(128, 1) → BN → ReLU → Upsample to 8 × 8
  - Concatenate all branches with encoder output:
  ppm_out = Concat([enc, *P*_1_, *P*_2_, *P*_3_, *P*_4_])
  - Bottleneck reduction: Conv2D(512, 1) → BN → ReLU
  Step 4: multi-scale feature fusion (MSFF)
  - Multi-scale branches:
  *F*_1_= Conv2D(*C*_1_, 1 × 1) → BN → ReLU
  *F*_2_= Conv2D(*C*_2_, 3 × 3) → BN → ReLU
  *F*_3_= Conv2D(*C*_3_, 3 × 3, dilation = 2) → BN → ReLU
  - Concatenation: *F*_cat_ = [*F*_1_∥*F*_2_∥*F*_3_]
  - Channel attention: att = σ(*W*_2_δ(*W*_1_GAP(*F*_cat_)))
  - Residual projection: *W*_*r*_*F*_in_= Conv2D(*C*, 1 × 1) → BN
  - Residual fusion: *F*_out_ = *W*_*r*_*F*_in_+(att⊙*F*_cat_)
  Step 5: decoder 
  Stage 1 (8 → 16): Up_2_ → Concat(MSFF(skip4)) → Conv2D(512)
  Stage 2 (16 → 32): Up_2_ → Concat(MSFF(skip3)) → Conv2D(256)
  Stage 3 (32 → 64): Up_2_ → Concat(MSFF(skip2)) → Conv2D(128)
  Stage 4 (64 → 128): Up_2_ → Concat(MSFF(skip1)) → Conv2D(64)
  Stage 5 (128 → 256): Up_2_ → Conv2D(32)
  Final output: Ŷ = Conv2D(1, 1 × 1, sigmoid)(*X*)

## 3 Evaluation metrics

To evaluate model performance, Intersection over Union (IoU), Dice (Dice Coefficient), Average Symmetric Surface Distance (ASSD), Hausdorff Distance 95th Percentile (HD95), and Floating Point Operations (flops) were used. The IoU is used to measure the overlap between the ground truth and prediction mask as the ratio of intersection to union.


$$\begin{array}{l} \text{IoU}=\frac{TP}{TP+FP+FN} \end{array}$$ (9)

In Equations 9, 10 *TP* denotes True Positives, *FP* denotes False Positives, and *FN* denotes False Negatives respectively.

The dice is used to measure segmentation overlap with emphasis on true positives.


$$\begin{array}{l} \text{Dice}=\frac{2TP}{2TP+FP+FN} \end{array}$$ (10)

HD95 measures the boundary distance between the ground truth and the predicted mask at the 95th percentile, thereby reducing outlier sensitivity.


$$\begin{array}{l} HD_{95}=HD_{95}\left(A,B\right) \end{array}$$ (11)

In Equation 11, *A* and *B* denote the predicted and ground truth segmentation boundaries, and *HD*_95_(*A, B*) represents the 95th percentile Hausdorff distance between them. The ASSD measures the average distance between the ground truth and the prediction mask boundaries in both directions.


$$ASSD=\frac{1}{\left|S_{A}|+|S_{B}\right|}\left(\sum_{x\in S_{A}}d(x,S_{B})+\sum_{y\in S_{B}}d(y,S_{A})\right)$$ (12)

In Equation 12 *S*_*A*_ and *S*_*B*_ denote the sets of surface points of the predicted and ground truth segmentations, respectively, and *d*(*x, S*) represents the minimum Euclidean distance from point *x* to surface *S*. FLOPs measure the computational complexity of the model by calculating the number of operations required to process the data.


$$\begin{array}{l} \text{FLOPs}=2\times C_{in}\times K_{h}\times K_{w}\times C_{out}\times H_{out}\times W_{out} \end{array}$$ (13)

In Equation 13, *C*_*in*_ and *C*_*out*_ denote the number of input and output channels, respectively, *K*_*h*_ and *K*_*w*_ represent the kernel height and width, and *H*_*out*_ and *W*_*out*_ denote the output feature map dimensions. All evaluation metrics were computed at the slice level and averaged across all slices. The evaluation was performed in a 2D slice-wise manner. To provide a patient-level perspective, slice-wise metrics were aggregated per patient by averaging across all valid slices belonging to each subject. Voxel spacing metadata was not consistently available across both datasets, and resizing to 256 × 256 pixels further precluded reliable physical unit computation. Therefore, boundary-based metrics, such as HD95 and ASSD, were computed in pixel units rather than physical units (mm). These metrics were calculated using the resized predictions and ground-truth masks. Therefore, comparisons with studies reporting results in millimeters should be made with caution. All experiments were repeated thrice using different random seeds (42, 21, and 7), and the results are reported as the mean ± standard deviation to assess result stability.

## 4 Result and discussion

The PSP-EffB0-MSFF model was developed to improve segmentation performance while maintaining a balance between accuracy and computational cost. An ablation study was conducted to evaluate the contribution of each component in the architecture.

As shown in Table 4 (slice-level results), for CirrMRI600+ dataset, the baseline PSPNet model with a ResNet50 backbone achieved a Dice score of 0.81 ± 0.233 with a computational cost of 12.85 GFLOPs. Replacing the backbone with EfficientNetB0 significantly reduced the computational cost to 3.17 GFLOPs, although a slight decrease in the Dice score to 0.79 ± 0.26 was observed. Adding skip connections improved the Dice score to 0.919 ± 0.16, indicating better preservation of spatial information. The inclusion of the MSFF module further increased the Dice score to 0.922 ± 0.15. Although this improvement is relatively small, it remained consistent across experiments. From a computational perspective, the skip-only model required 17.72 GFLOPs. Incorporating the MSFF module reduced the computational cost compared to the skip-only configuration while maintaining similar segmentation performance. However, the overall computational cost remained higher than that of the EfficientNetB0 baseline, indicating a trade-off between improved accuracy and computational complexity. This suggests that the MSFF module mainly refines local feature representation, leading to slight improvements in boundary alignment rather than large changes in overlap-based metrics such as Dice.

**Table 4:** Ablation study for CirrMRI600+ dataset.

Method	IoU	Dice	HD95	ASSD	FLOPs
PSPNet with ResNet50	0.734 ± 0.254	0.81 ± 0.233	11.25 ± 13.57	3.361 ± 4.37	12.85G
PSPNet with EfficientNetB0	0.715 ± 0.261	0.79 ± 0.26	11.83 ± 13.55	3.63 ± 3.6	3.17G
PSPNet + EffB0 + skip	0.88 ± 0.17	0.919 ± 0.16	6.2 ± 12.39	1.59 ± 2.49	17.72G
PSPNet + EffB0 + skip + MSFF	0.88 ± 0.17	0.922 ± 0.15	5.924 ± 11.36	1.56 ± 2.19	14.91G

In addition to the Dice score, improvements were observed in the boundary-sensitive metrics. Specifically, the reductions in HD95 and ASSD across both datasets indicate slight refinement in boundary alignment with the MSFF module. These improvements suggest that the MSFF enhances fine-grained feature representation, particularly in regions where the liver boundary is adjacent to surrounding structures. Furthermore, the MSFF module reduces the computational cost compared with the skip-only configuration, indicating more efficient feature utilization. As shown in Table 5 (slice-level results), a similar trend was observed for DLDS dataset. A comparison with prior work on the DLDS dataset was not performed, as many existing studies report distance-based metrics (HD95 and ASSD) in physical units (mm).

**Table 5:** Ablation study for DLDS dataset.

Method	IoU	Dice	HD95	ASSD	FLOPs
PSPNet with ResNet50	0.844 ± 0.121	0.909 ± 0.095	8.431 ± 7.769	2.377 ± 1.497	12.85G
PSPNet with EfficientNetB0	0.835 ± 0.126	0.904 ± 0.099	8.947 ± 7.673	2.509 ± 1.499	3.17G
PSPNet + EffB0 + skip	0.937 ± 0.062	0.952 ± 0.049	2.612 ± 6.421	0.754 ± 0.994	17.72G
PSPNet + EffB0 + skip + MSFF	0.943 ± 0.059	0.955 ± 0.038	2.341 ± 4.921	0.681 ± 0.996	14.91G

The baseline model achieved a Dice score of 0.909 ± 0.095. Replacing the backbone with EfficientNetB0 reduced the computational cost but slightly decreased performance. The addition of skip connections significantly improved the Dice score to 0.952 ± 0.049, highlighting their role in preserving spatial information. Incorporating the MSFF module further increased the Dice score to 0.955 ± 0.038. Although the improvement was modest, it consistently enhanced segmentation performance, particularly near object boundaries. Overall, skip connections contributed the most to performance gains, while the MSFF module provided additional refinement. Although the improvement from MSFF was incremental, it remains relevant for tasks requiring precise boundary delineation, which is important for clinical interpretation. The results demonstrate an accuracy–complexity trade-off, where improved segmentation performance is achieved at the cost of increased computational complexity compared to the baseline. As shown in Table 6, the proposed model showed competitive performance within the current experimental setup. However, these comparisons should be interpreted with caution because differences in datasets, imaging modalities, preprocessing strategies, and evaluation protocols may influence the reported performance. Therefore, the results are presented in a general context rather than a direct comparison.

**Table 6:** Performance comparison on the CirrMRI600+ dataset.

Method	IoU	Dice	HD95	ASSD	Dataset
UNet (26)	0.6772	0.6900	38.22	10.11	CirrMRI600+
AttentionUNet (26)	0.7089	0.7288	36.19	9.28	CirrMRI600+
nnUnet-2D (26)	0.7229	0.7418	34.56	8.78	CirrMRI600+
Trasunet (26)	0.7219	0.7457	31.11	8.66	CirrMRI600+
Synergynet (26)	0.7383	0.7592	30.94	7.55	CirrMRI600+
MedSegDiff (26)	0.7489	0.7667	30.89	7.34	CirrMRI600+
PSP + EffB0 + skip + MSFF (ours)	0.879 ± 0.1674	0.922 ± 0.1524	5.924 ± 11.358	1.559 ± 2.198	CirrMRI600+

**Figure 5:**
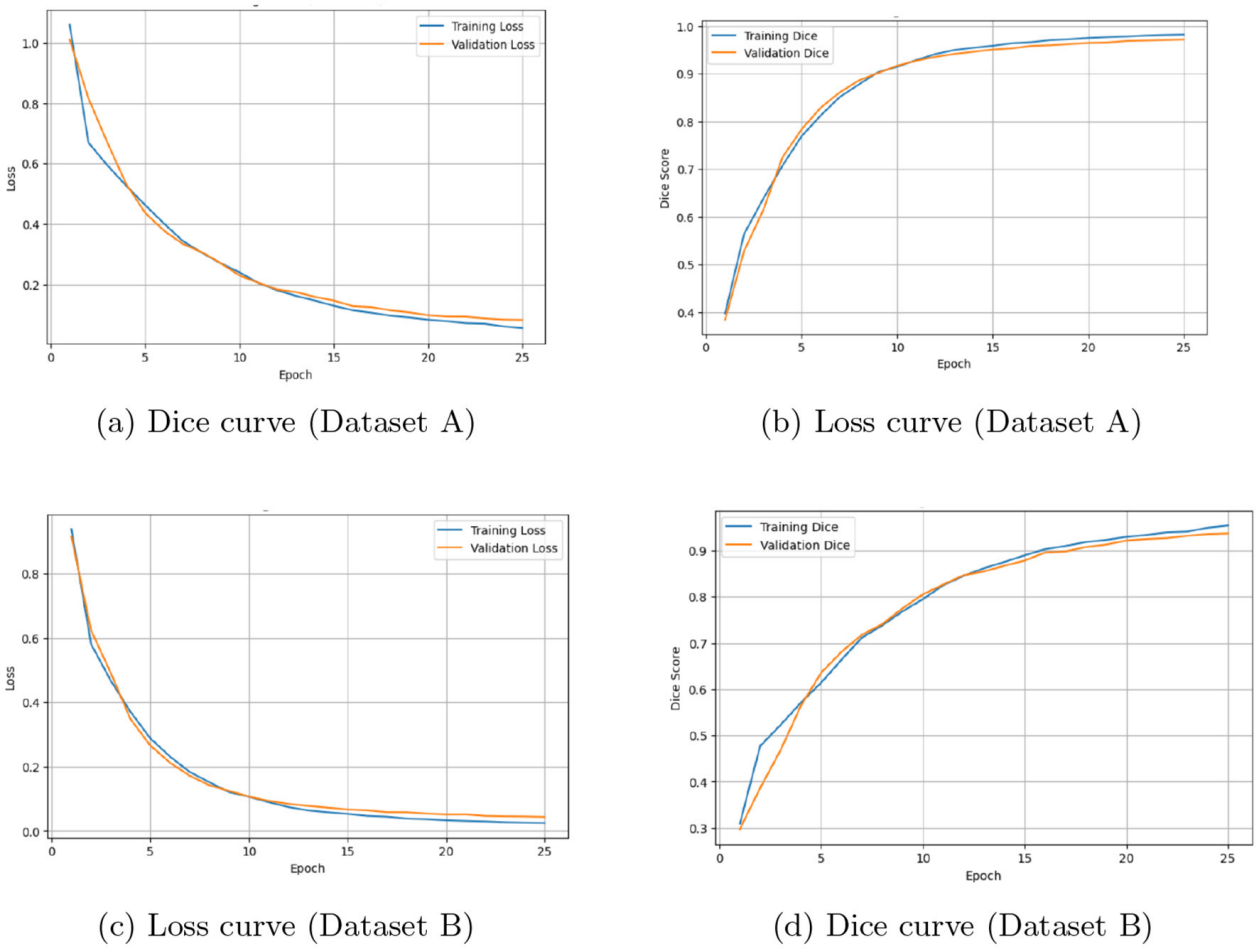
Training and validation Dice and loss curves for Dataset A and Dataset B. **(a)** Dice curve (Dataset A). **(b)** Loss curve (Dataset A). **(c)** Loss curve (Dataset B). **(d)** Dice curve (Dataset B).

The predicted segmentations largely aligned with the ground truth, with minor discrepancies observed near boundary regions where the liver was adjacent to surrounding structures. From a clinical perspective, accurate liver segmentation supports volumetric analysis, treatment planning, and disease monitoring, contributing to more reliable quantitative assessments. This study has several limitations. The model operates on 2D slices, which means that full 3D spatial relationships across slices are not explicitly captured and may affect volumetric consistency. In addition, the boundary-based metrics (HD95 and ASSD) were computed in pixel units because consistent voxel spacing information was not available, which limits their direct clinical interpretation. During training, only a fixed number of slices were selected from each patient to keep the computation manageable; however, this may not fully utilize all available volumetric information. Moreover, cross-dataset generalization was not evaluated, as training and testing were performed separately on each dataset. Although the proposed model was evaluated across multiple runs, ablation experiments were conducted using a single run because of computational constraints; therefore, the observed improvements should be interpreted as indicative trends rather than statistically validated differences. Experiments for the proposed model were repeated across three independent runs using fixed random seeds (42, 21, and 7) to assess result stability, whereas ablation studies were conducted using a single run owing to computational constraints. Similar performance trends observed across both datasets suggest consistent model behavior. The model was trained using a combination of binary cross-entropy (BCE) and Dice loss. BCE penalizes pixel-level misclassifications, whereas the Dice loss directly optimizes the overlap between the predicted and ground truth masks and addresses class imbalance. During training, the loss decreased rapidly in the initial epochs and stabilized by epoch 25 for both datasets, as illustrated in Figure 5.

**Figure 6:**
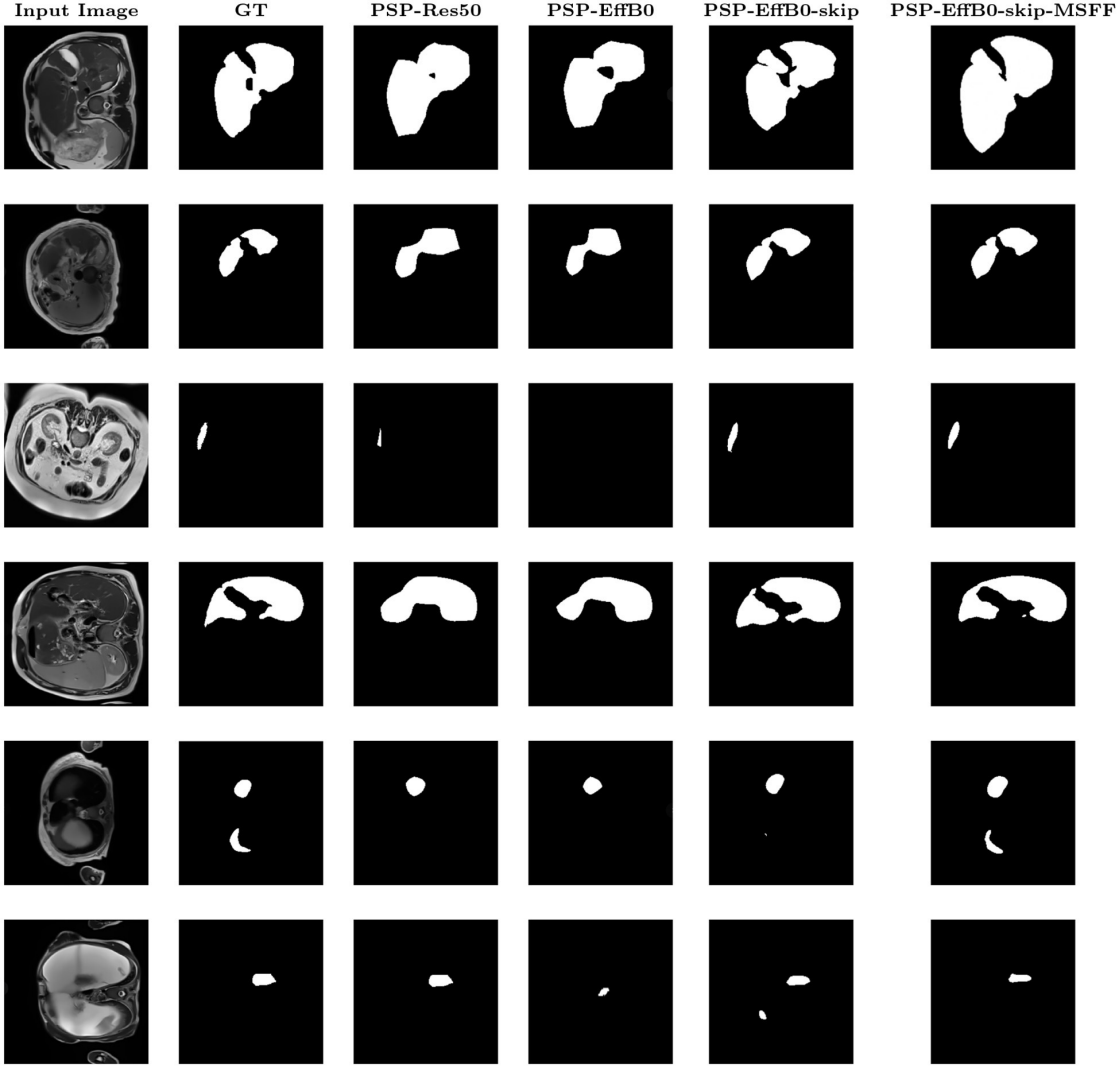
Qualitative visualization of PSPNet variants on Dataset A.

**Figure 7:**
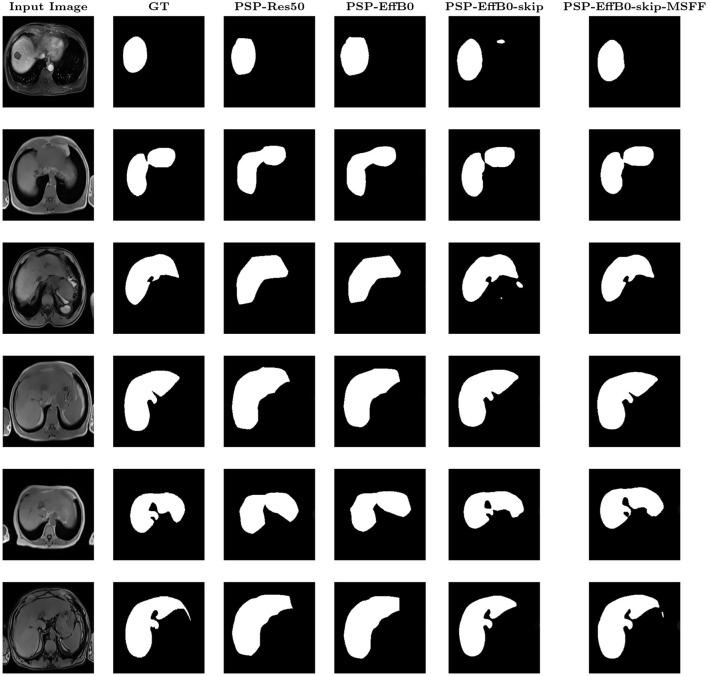
Qualitative visualization of PSPNet variants on Dataset B.

The patient-level results, obtained by aggregating slice-wise predictions for each subject, are presented in Table 7. When the average Dice is computed across all slices, including background-only slices, the patient-level Dice is lower than the slice-level Dice computed on liver-containing slices. Ablation studies were conducted at the slice level to analyze the contribution of individual components. Although the model operates on 2D slices, patient-level evaluation provides a more clinically relevant assessment of segmentation performance. The training and validation curves remained closely aligned, indicating stable convergence with minimal overfitting. Figures 6, 7 show the qualitative results for Datasets A and B, respectively, where the MSFF module showed relatively improved boundary delineation compared to other configurations, as observed in the qualitative results.

**Table 7:** Performance of the proposed model on CirrMRI600+ and DLDS datasets (mean ± standard deviation over three runs).

Method	IoU	Dice	HD95	ASSD	FLOPs
CirrMRI600+	0.86 ± 0.01	0.90 ± 0.02	6.20 ± 0.60	9.80 ± 1.50	14.91*G*
DLDS	0.905 ± 0.038	0.913 ± 0.09	7.31 ± 3.91	2.66 ± 3.06	14.91*G*

## 5 Conclusion

U-Net based architectures are widely used for medical image segmentation. In this study, the proposed PSP-EffB0-MSFF model demonstrated competitive performance on the CirrMRI600+ dataset and consistent segmentation performance on the DLDS under the current experimental setup. The inclusion of skip connections plays a major role in improving the segmentation performance, whereas the MSFF module provides additional refinement. Although the improvement in the Dice score was modest, consistent reductions in distance-based metrics (HD95 and ASSD) indicated slight refinement in boundary alignment, particularly in regions where the liver was adjacent to the surrounding structures. This suggests that the MSFF enhances local feature representation rather than producing large global performance gains. EfficientNetB0 was chosen as the backbone due to its balance between computational cost and segmentation performance, enabling a practical trade-off between accuracy and efficiency. Future work will focus on extending the proposed approach to other imaging modalities, such as CT and ultrasound, exploring alternative backbone architectures, incorporating volumetric evaluation metrics, and performing standardized comparisons on the DLDS dataset using physical unit-based metrics, as voxel spacing metadata was not consistently available in the current dataset.

## Data Availability

The original contributions presented in the study are included in the article/supplementary material, further inquiries can be directed to the corresponding author.
